# Suicide Models and Treatment Models Are Separate Entities. What Does It Mean for Clinical Suicide Prevention?

**DOI:** 10.3390/ijerph18105301

**Published:** 2021-05-17

**Authors:** Konrad Michel

**Affiliations:** University Hospital of Psychiatry, University of Bern, CH-3008 Bern, Switzerland; konrad.michel@upd.unibe.ch

**Keywords:** suicide prevention, suicide models, psychological treatments, suicide risk assessment

## Abstract

Theoretical models of suicide are based on risk factors associated with suicide, such as psychiatric pathology, genetics, epigenetics, functional brain disorders, and impaired decision making. In current clinical practice, the predominant risk model is the medical model, which posits that treating suicide risk is primarily a matter of treating psychiatric disorders. However, even comprehensive risk factor models cannot overcome the basic problem that, by their nature, they cannot accommodate the suicidal person’s psychological experience of suicidality. Risk factor models do not translate into effective treatment models. Suicide risk is highly personal and fluid, and is related to individual vulnerabilities and to person-specific events triggering suicidal thoughts and actions. Clinicians need treatment models that are meaningful to suicidal patients. Understanding the single person’s suicidality requires a patient-centered approach. Therapeutic interventions that effectively reduce the risk of suicidal behavior have been developed from general principles of psychotherapy. Therapy process factors associated with effective therapies are working alliance, validation of the individual patient’s thoughts and feelings, active treatment engagement. Psychological therapies need patients who are active participants in a collaborative working relationship between therapist and patient. The goal must be to jointly develop a meaningful understanding of the suicidal crisis. In view of the limited personal resources in health care systems it is important that effective therapies are brief and effective. Future research must aim to improve our understanding of the factors involved in effective therapies in order to optimize treatments for individuals at risk. This may also include the integration of biological risk factors in psychological treatment models.

## 1. Introduction

Suicide is a highly complex phenomenon of human behavior. Most theoretical models attempt to explain suicide by reducing suicidal behavior to risk factors. In 1897 Durkheim, with “Le Suicide”, put forward a sociological model of suicide, in which problems with social integration were identified as the main causes of suicide [1]. These included risk factors such as being single, having higher levels of education, and being of Protestant denomination. In the second half of last century, theoretical models focused predominantly on medical risk factors. The classical studies by Robins [2], Conwell [3], and Harris and Barraclough [4] consistently found that in more than 90 percent of suicides the criteria for a psychiatric diagnosis were fulfilled. Since then, the association between suicide and mental illness has largely determined the practice of clinical suicide prevention. The illness model of suicide has later received support from other studies [5,6]. In recent years, comprehensive models of suicide have been put forward [7,8]. Yet so far, no theoretical model has successfully been translated into a clinical treatment protocol with evidence of effectively reducing suicide.

In contrast, most psychological therapies have been developed from general psychotherapy research and practice, with a strong emphasis on therapy process factors such as treatment engagement and working alliance. By their nature, they focus on the person’s individual, subjective experience of a suicidal crisis. Today, there is compelling evidence that person-centered psychological treatments can significantly reduce suicidal behavior, particularly for patients with a history of attempted suicide.

## 2. The Limitations of Theoretical Suicide Models

In suicide literature, studies about suicide risk factors abound. Typical risk factors encompass psychiatric disorders and physical illness, personal characteristics such as a history of attempted suicide, relationship and work problems, separation and loss, isolation, etc. Biological risk factors include genetic and epigenetic factors and certain personality traits. The biomedical model posits that suicide is mostly a consequence of psychiatric pathology [9,10], and inadequate treatment of depression [5,11,12]. Training schemes for primary care physicians and fight depression campaigns have been developed [13,14]. Yet, over the years, the results have been sobering. In spite of the widespread efforts to train health professionals, affective disorders in primary care continue to be underdiagnosed. And, in spite of population-based campaigns, too many clinically depressed individuals—predominantly men—do not seek help. Bertolote and co-authors from the Department of Mental Health and Substance Dependence of the WHO in Geneva [15] took a worldwide view on the topic, assuming that antidepressants would be effective in treating depression in about 52% (a figure taken from the World Health Report 2001) and that some 50% of people with depression were correctly diagnosed and treated. The impact on suicide rates was estimated to be about 7.8%, which would reduce suicide rates from a world average of 15.1 per 100,000 to 13.9 per 100,000. Furthermore, the evidence for the effect of antidepressant treatment on suicide is controversial [16,17,18,19]. Randomized trials of psychopharmacological interventions usually exclude patients with a history of suicidal ideation or behavior and those who are actively suicidal, limiting the generalizability of the findings [20].

In an attempt to improve current models of suicide, comprehensive models including different categories of risk factors have been developed. In Mann’s diathesis-stress model [7] diathesis is understood as a constitutional or acquired vulnerability (for instance, impulsive and aggressive traits) as risk factors for suicide. Stress factors are assumed to be internal (e.g., depression) or external (e.g., problems with relationships or employment). Later, Mann and Rizk [21] updated the stress-diathesis model with a “brain-centric model of suicidal behavior”, and added recommendations for suicide prevention. The biopsychosocial model by Turecki et al. [22] differentiates between distal, developmental, and proximal factors. Risk factors include early life adversities, epigenetic changes, personality traits such as impulsive aggression, hopelessness and unbearable psychological pain, etc. The integrated motivational-volitional model of suicidal behavior [8] includes three dimensions: the biopsychosocial context as premotivational phase, the emergence and maintenance of ideation as motivational phase, and the transition to suicidal behaviors in the volitional phase. In a clinical application this model on suicide attempters did not reduce the risk of reattempts [23]. In Joiner’s interpersonal theory of suicide [24,25] individuals engage in serious suicidal behavior as a result of interpersonal psychological states, such as perceived burdensomeness (being a burden to others), and thwarted belongingness (feelings of alienation). It is assumed that these feelings lead to the belief that one’s death is worthwhile to others, and to the desire to die. Repeated experiences of pain, often by self-injury, are seen as a way of acquiring the capability for suicide. To our knowledge, the interpersonal theory has not been tested in a treatment study.

These models normally assume a trajectory from suicide ideation to suicide action, that is, a developmental pathway from psychological stressors to ideation and from ideation to action, similar to a linear pathogenetic model in somatic medicine. For instance, in diabetes mellitus type 2, a causal factor is insulin resistance due to receptor abnormalities, leading to hyperglycemia, polyuria, inactivity, obesity, and, as long-term consequences to vascular lesions with coronary heart disease, retinopathy, nephropathy, and neuropathy–the concept of a progressing pathology from a molecular level to the long-term clinical consequences. However, suicide as a behavior does not fit into a linear illness model. Theoretical models by their nature cannot accommodate the very personal psychology of the individual considering suicide. The emotional valence and the meaning of a specific psychosocial stressor are related to a person’s, often biographically determined, vulnerability (for instance early trauma). Typically, suicidal risk waxes and wanes. Rudd [26] formulated The Fluid Vulnerability Theory, a concept which assumes a long-term baseline risk that varies from individual to individual, and a short-term risk that is highly determined by aggravating factors active for limited periods of time (hours, days, weeks). Bryan and Rudd [27] argued that the “ideation-to-action framework” with suicidal thoughts as starting point and suicide or suicide attempt as an end point has little to do with the clinical reality of suicidal individuals.

In clinical practice, risk factor models are used for suicide risk assessment. However, studies on the predictive value of suicide risk scales have yielded sobering results [28,29]. Suicide risk scales with multiple risk factors do not add to the statistical strength [30]. A meta-analysis on 365 studies concluded that the ability of risk factor models to predict suicide has not significantly improved over 50 years of research, and recommended a shift in focus from risk factors to machine-learning-based risk algorithms [31]. However, considering the very personal and specific individual psychological vulnerability of each individual, it is difficult to imagine how novel risk factors and machine learning involving large numbers (>100) of risk factors [32] will resolve the underlying conceptual problem. No model can foresee the time and nature of intra- and interpersonal conflicts that may (or may not) trigger suicidal behavior. By their nature, risk factor models can only provide information on mid-term or long-term risk, but not on the risk in a time frame of hours and days. In clinical practice, risk-factor-based suicide screening can be used as an initial step leading to a person-centered risk assessment [33,34].

In recent years, several experts have argued that new approaches in clinical suicide prevention are needed [35,36,37,38]. The insight is growing that current practices will not get us any further in significantly reducing suicide.

## 3. The Promise of Suicide-Specific, Person-Centered Psychological Treatments

In contrast to risk-factor models of suicide, psychological treatment models focus on the person’s individual and “subjective” experience of a suicidal crisis. These models have usually been developed from general principles of psychotherapy. Key elements are the concepts of therapeutic relationships and alliances. The therapeutic alliance is best defined as “the active and purposeful collaboration between patient and therapist” [39]. There is a wealth of studies, most of them published in the second half of the last century, focusing on the characteristics of an effective therapeutic relationship [40,41,42]. In a meta-analysis of 201 psychotherapy research reports, Horvath et al. [43] found a strong relationship between alliance and the therapy outcome. They concluded that alliance is best understood as a measure of how well the therapist and client work together in therapy as a collaborative enterprise. In their contextual analysis, Wampold and Imel [44] stressed the importance of empathy, goal consensus, and collaboration to achieve change in psychotherapy.

Considering the high rates of non-attendance and treatment dropout of suicidal patients [45,46,47], treatment engagement is of paramount importance [48]. A prerequisite for treatment engagement is that the therapy model and content are meaningful to the patient. The therapist’s aim must be to understand the person’s subjective inner experience related to the suicidal crisis [49]. Patient-rated measures of therapeutic alliance can be an indicator of how meaningful a therapy concept for this group of patients is. In a review, Dunster-Page et al. [50] analyzed the effect of therapeutic alliance on suicidal ideation and behavior. Therapy models included Dialectical Behavior Therapy (DBT), Cognitive Behavior Therapy (CBT, BCBT), the Attempted Suicide Short Intervention Program (ASSIP), and dynamic psychotherapy. The authors concluded that an alliance with either a therapist, care coordinator, or mental health team has a significant impact on a patient’s suicidality. For example, assessment tools for therapeutic alliance used by Gysin-Maillart et al. [51,52] and Bryan et al. [53] were the Helping Alliance Questionnaire [54], and the Working Alliance Inventory [55]. Both studies found an inverse relationship between alliance and suicidal ideation during follow-up.

Several treatment studies have focused on ED-based interventions, with usually short follow-up periods. The ED setting is particularly important, as large numbers of at-risk individuals use emergency services [56]. From a meta-analysis, Inagaki et al. [57] concluded that for patients admitted to ED active contact and follow-up may reduce the risk of a repeat suicide attempt up to 12 months, although the results are mixed [58].

The main psychological treatments based on manualized treatment protocols, with at least one well-conducted study that yielded significant effects in reducing suicidal behavior in high-risk patients, are Cognitive Behavioral Therapy (CBT, BCBT), Dialectical Behavioral Therapy (DBT), the Attempted Suicide Short Intervention Program (ASSIP), and the Collaborative Management of Suicidality (CAMS). The lengths of follow-up in these randomized studies range from six months [59] to 18 [60] and 24 months [61,62].

In Cognitive Behavioral Therapy [63] therapist and patient together explore the patients’ core beliefs and automatic thoughts, to develop individual (often homework-based) goals. The emphasis is on the collaborative exploration of a person’s cognitions (the private meaning assigned by the individual) and the “suicidal belief system”. The therapist is an active and engaged expert, focusing on symptom management and crisis resolution, skill-building, and personality development. A central element is the concept of the suicidal mode, based on a model of crisis as a time-limited mental state. Modes encompass cognitions, emotions, physiological symptoms, and behavior patterns, and are usually triggered, and typically have an on/off mechanism [64,65]. Dialectical Behavioral Therapy combines change strategies from cognitive-behavioral therapy with acceptance strategies, an active process, demonstrated through the use of validation strategies [66]. The main goal is to teach the patient skills to regulate emotions and improve relationships with others. The therapist’s role is characterized by the tension between accepting the patient’s inner experience at a given moment, and simultaneously getting the patient to change maladaptive behavioral patterns. The therapeutic relationship is characterized by an ongoing expression of acceptance on the part of the therapist towards the patient [67]. The Attempted Suicide Short Intervention Program is a three-session patient-centered brief therapy that is aimed to maximize the patients’ active participation and treatment engagement [68]. Therapy components are a narrative interview, video-recorded (1st session), a video-playback of the patient’s narrative in which therapist and patient collaboratively reflect about the patient’s personal needs and vulnerabilities, trigger events, and the suicide action (2nd session), a collaborative psychoeducational handout, and a jointly developed case conceptualization with individual warning signs and safety strategies (3rd session). The face-to-face sessions are followed by an active outreach element, that is, regular, personalized letters to the participants for 24 months, as a continuation of a minimal therapeutic relationship and a reminder of the work done. In an RCT with 120 patients, the risk of suicide reattempts in the treatment group was reduced by 80% [51]. In the Collaborative Assessment and Management of Suicidality [69] alliance is achieved by engaging the suicidal patient as an active participant in the assessment of the suicidal risk and by collaborating with the patient as a co-author of the suicide-specific treatment plan. The patient’s view is the “gold standard” for risk assessment. The focus of the Suicide Status Form SSF [68] is primarily on the patient’s psychological pain and suffering. The therapist serves as a consultant, coach, and co-author.

What are the therapy process factors that make psychological treatments for suicidal individuals effective? Rudd and colleagues [70] identified common elements of treatments that work, distilled from a review of available randomized clinical trials targeting suicidality. One such element was providing patients with simple and understandable models for suicidality. A second element is facilitating hope, which is expected to have positive implications in motivation, commitment, and overall treatment compliance. Others [71,72] have identified common elements of effective therapies, such as a clear treatment framework, active engagement and participation of the therapist and patient, and behavioral validation. According to Self-Determination Theory adopting new goals and a new behavior requires intrinsic motivation, and this happens when clients feel listened to, valued, and understood by their therapist [73]. The therapeutic attitude should be noncoercive and nonthreatening, creating an atmosphere that is empathic, non-judgmental, and supportive of the patient’s concerns [48,74]. McCabe et al. [75] from a meta-analysis concluded that effective treatments are associated with changes in behavior (suicide attempts) but do not appear to reduce suicidal ideation. Therapeutic interventions that directly address suicidal thoughts and behavior are particularly effective [76]. CBT, DBT, and ASSIP have highly structured therapy protocols with declared therapy goals, fostering insight, helping patients to anticipate future suicidal crises, and recognizing their personal warning signs. The ASSIP treatment model is built on the theory that suicide is a goal-directed action, which, as in everyday life, is explained through stories [77]. The first session is fully reserved for the patient’s narrative of the suicidal crisis. The goal is a collaborative case formulation, which includes personal warning signs and safety strategies.

In interviewing suicidal patients, clinicians have a dual role: They must be interested and attentive listeners to the suicidal person’s story and try to understand the suicidal development, but they must also conduct a clinical assessment of medical and personal risk factors. However, interviews with suicidal patients should put the person-centered narrative approach first, with the interviewer in the not-knowing position, inviting the patient to explain his or her reasons for the suicidal behavior.

## 4. Risk Factor Models May Complement Psychological Treatment Models

There are areas where general suicide models and person-centered treatment models meet. For instance, the concept of epigenetics has helped to understand the psychological sequela of early traumatic experiences ([78,79]. Neurobiological research into problem-solving can help therapists in dealing with patients’ deficits in decision-making [80]. Similarly, the insight that problems of emotion regulation and impulse control have a neurophysiological basis has been incorporated in the Biosocial Theory of DBT, where biological vulnerabilities and individual factors (like temperament) interact with environmental factors [81]. We [82] have attempted to bridge the gap between the suicidal patients’ personal experience by measuring the suicide-script-driven neural activation with functional imaging. The diathesis concept of neurobiological risk factors (early trauma, problems with emotion control, etc.) suggests that realistic treatment goals may not be to “cure” long-term suicide risk, but, above all, for patients to develop and acquire effective coping strategies, to compensate for biological vulnerabilities. In our clinical work, we found that including biological aspects as part of psychoeducation, can help to reduce shame and guilt related to impulsive behavior [83]. The model of an overresponsive stress system as a trait-like condition can help patients to understand why it is important to develop cognitive and behavioral skills to cope with future suicide-specific stressful situations. This may also explain to patients the rationale for combining pharmacological treatments (for instance with lithium) with psychological treatments, where indicated.

## 5. Conclusions

Suicide is a complex and highly multifactorial phenomenon. Theoretical models of suicide are important and have contributed to our knowledge of factors involved in suicidal behavior. Yet, the expectation that comprehensive suicide models will lead to effective strategies in reducing suicide has not been fulfilled. We must abandon the idea that even more refined theoretical models will finally result in effective therapies for suicidal individuals. In clinical suicide prevention, we must look beyond risk factor models. Suicidal behavior is inherently individual and personal. This does not preclude psychiatric assessment and treatment of associated psychiatric disorders as risk factors. However, engaging patients in collaborative treatment requires a patient-oriented approach and a strong therapeutic relationship. A meaningful therapeutic discourse needs two protagonists who share a common ground. This stands in contrast to an illness-based approach to the suicidal individual where the treatment of mental disorders has priority. As long as clinicians see suicidal patients as passive objects and the clinician as the all-knowing expert we shall not move forward in reducing suicide. Clinical suicide prevention needs to integrate more psychotherapeutic concepts in the treatment of patients at risk of suicide. There are now several effective psychological treatments, but, so far, they play a marginal role in the provision of care for suicidal patients. A major problem is a huge discrepancy between the number of suicide attempts and the limitations of the health care systems in the provision of therapies for suicidal patients. However, certain hope comes from studies that show that brief and specific therapies can be highly effective. Ideally, they have a clear structure, are easy to understand for patients, define clear treatment goals, and directly address suicidal thoughts and behavior.

## Data Availability

Not applicable.

## References

[1] Durkheim E. (1897). Le Suicide: Etude de Sociologie (Suicide: A Sociological Study).

[2] Robins E., Murphy G.E., Wilkinson R.H., Gassner S., Kayes J. (1959). Some Clinical Considerations in the Prevention of Suicide Based on a Study of 134 Successful Suicides. Am. J. Public Health Nations Health.

[3] Conwell Y., Duberstein P.R., Cox C., Herrmann J.H., Forbes N.T., Caine E.D. (1996). Relationships of age and axis I diagnoses in victims of completed suicide: A psychological autopsy study. Am. J. Psychiatry.

[4] Harris E.C., Barraclough B. (1997). Suicide as an outcome for mental disorders. Br. J. Psychiatry.

[5] Isometsä E.T., Aro H.M., Henriksson M.M., Heikkinen M.E., Lönnqvist J.K. (1994). Suicide in major depression in different treatment settings. J. Clin. Psychiatry.

[6] Bostwick J.M., Pankratz V.S. (2000). Affective Disorders and Suicide Risk: A Reexamination. Am. J. Psychiatry.

[7] Mann J.J., Waternaux C., Haas G.L., Malone K.M. (1999). Toward a clinical model of suicidal behavior in psychiatric patients. Am. J. Psychiatry.

[8] O’Connor R.C. (2011). The integrated motivational-volitional model of suicidal behavior. Crisis.

[9] Miles C.P. (1977). Conditions predisposing to suicide: A review. J. Nerv. Ment. Dis..

[10] Isacsson G. (2000). Suicide prevention--a medical breakthrough?. Acta Psychiatr. Scand..

[11] Oquendo M.A., Malone K.M., Ellis S.P., Sackeim H.A., Mann J.J. (1999). Inadequacy of antidepressant treatment for patients with major depression who are at risk for suicidal behavior. Am. J. Psychiatry.

[12] Suominen K.H., Isometsä E.T., Henriksson M.M., Ostamo A.I., Lönnqvist J.K. (1998). Inadequate Treatment for Major Depression Both Before and After Attempted Suicide. Am. J. Psychiatry.

[13] Rutz W., Wålinder J., Eberhard G., Holmberg G., Von Knorring A.L., Von Knorring L., Wistedt B., Aberg-Wistedt A. (1989). An educational program on depressive disorders for general practitioners on Gotland: Background and evaluation. Acta Psychiatr. Scand..

[14] Hegerl U., Rummel-Kluge C., Värnik A., Arensman E., Koburger N. (2013). Alliances against depression—A community based approach to target depression and to prevent suicidal behaviour. Neurosci. Biobehav. Rev..

[15] Bertolote J.M., Fleischmann A., De Leo D., Wasserman D. (2003). Suicide and mental disorders: Do we know enough?. Br. J. Psychiatry.

[16] Van Praag H.M. (2003). A stubborn behaviour: The failure of antidepressants to reduce suicide rates. World J. Biol. Psychiatry.

[17] Simon G.E., Savarino J., Operskalski B., Wang P.S. (2006). Suicide Risk during Antidepressant Treatment. Am. J. Psychiatry.

[18] Stone M., Laughren T., Jones M.L., Levenson M., Holland P.C., Hughes A., Hammad T.A., Temple R., Rochester G. (2009). Risk of suicidality in clinical trials of antidepressants in adults: Analysis of proprietary data submitted to US Food and Drug Administration. BMJ.

[19] Braun C., Bschor T., Franklin J., Baethge C. (2016). Suicides and Suicide Attempts during Long-Term Treatment with Antidepressants: A Meta-Analysis of 29 Placebo-Controlled Studies Including 6,934 Patients with Major Depressive Disorder. Psychother. Psychosom..

[20] Fazel S., Runeson B. (2020). Suicide. N. Engl. J. Med..

[21] Mann J.J., Rizk M.M. (2020). A Brain-Centric Model of Suicidal Behavior. Am. J. Psychiatry.

[22] Turecki G., Brent D.A., Gunnell D., O’Connor R.C., Oquendo M.A., Pirkis J., Stanley B.H. (2019). Suicide and suicide risk. Nat. Rev. Dis. Prim..

[23] O’Connor R.C., Ferguson E., Scott F., Smyth R., McDaid D., Park A.-L., Beautrais A., Armitage C.J. (2017). A brief psychological intervention to reduce repetition of self-harm in patients admitted to hospital following a suicide attempt: A randomised controlled trial. Lancet Psychiatry.

[24] Joiner T.E. (2005). Why People Die by Suicide.

[25] Van Orden K.A., Witte T.K., Cukrowicz K.C., Braithwaite S.R., Selby E.A., Joiner T.E. (2010). The interpersonal theory of suicide. Psychol. Rev..

[26] Rudd M.D. (2006). Fluid vulnerability theory: A cognitive approach to understanding the process of acute and chronic risk. Cognition and Suicide: Theory, Research, and Therapy.

[27] Bryan C.J., Rudd M.D. (2016). The importance of temporal dynamics in the transition from suicidal thought to behavior. Clin. Psychol. Sci. Pract..

[28] Chan M.K.Y., Bhatti H., Meader N., Stockton S., Evans J., O’Connor R.C., Kapur N., Kendall T. (2016). Predicting suicide following self-harm: Systematic review of risk factors and risk scales. Br. J. Psychiatry.

[29] Mulder R., Newton-Howes G., Coid J.W. (2016). The futility of risk prediction in psychiatry. Br. J. Psychiatry.

[30] Large M., Kaneson M., Myles N., Myles H., Gunaratne P., Ryan C. (2016). Meta-Analysis of Longitudinal Cohort Studies of Suicide Risk Assessment among Psychiatric Patients: Heterogeneity in Results and Lack of Improvement over Time. PLoS ONE.

[31] Franklin J.C., Ribeiro J.D., Fox K.R., Bentley K.H., Kleiman E.M., Huang X., Musacchio K.M., Jaroszewski A.C., Chang B.P., Nock M.K. (2017). Risk factors for suicidal thoughts and behaviors: A meta-analysis of 50 years of research. Psychol. Bull..

[32] Ribeiro J.D., Franklin J.C., Fox K.R., Bentley K.H., Kleiman E.M., Chang B.P., Nock M.K. (2016). Letter to the Editor: Suicide as a complex classification problem: Machine learning and related techniques can advance suicide prediction—A reply to Roaldset (2016). Psychol. Med..

[33] Michel K. (2020). Suicide Risk Assessment Must be Collaborative. Acta Sci. Med. Sci..

[34] Bjureberg J., Dahlin M., Carlborg A., Edberg H., Haglund A., Runeson B. (2021). Columbia-Suicide Severity Rating Scale Screen Version: Initial screening for suicide risk in a psychiatric emergency department. Psychol. Med..

[35] De Leo D. (2002). Why are we not getting any closer to preventing suicide?. Br. J. Psychiatry.

[36] Stanley B., Mann J.J. (2020). The Need for Innovation in Health Care Systems to Improve Suicide Prevention. JAMA Psychiatry.

[37] Espeland K., Hjelmeland H., Knizek B.L. (2021). A call for change from impersonal risk assessment to a relational approach: Professionals’ reflections on the national guidelines for suicide prevention in mental health care in Norway. Int. J. Qual. Stud. Health Well-Being.

[38] Michel K., Valach L., Gysin-Maillart A. (2017). A Novel Therapy for People Who Attempt Suicide and Why We Need New Models of Suicide. Int. J. Environ. Res. Public Health.

[39] Michel K., Michel K., Jobes D. (2010). General Aspects of Therapeutic Alliance. Building a Therapeutic Alliance with the Suicidal Patient.

[40] Horvath A.O., Gaston L., Luborsky L., Miller L.L.N., Barber J., Docherty J.P. (1993). The Therapeutic Alliance and Its Measures.

[41] Horvath A.O., Greenberg L.S. (1994). The Working Alliance: Theory, Research and Practice.

[42] Gaston L., Thompson L., Gallagher D., Cournoyer L.G., Gagnon R. (1998). Alliance, technique, and their interactions in predicting outcome of behavioural, cognitive, and brief dynamic therapy. Psychother. Res..

[43] Horvath A.O., Del Re A.C., Flückiger C., Symonds D.B. (2011). Alliance in individual psychotherapy. Psychotherapy.

[44] Wampold B.E., Imel Z.E. (2015). Counseling and Psychotherapy. The Great Psychotherapy Debate: The Evidence for What Makes Psychotherapy Work.

[45] Granboulan V., Roudot-Thoraval F., Lemerle S., Alvin P. (2001). Predictive factors of post-discharge follow-up care among adolescent suicide attempters. Acta Psychiatr. Scand..

[46] Möller H.J., Ferrari G., Bellini M., Crepet P. (1990). Evaluation of aftercare strategies. Suicidal Behaviour and Risk Factors.

[47] Monti K., Cedereke M., Öjehagen A. (2003). Treatment Attendance and Suicidal Behavior 1 Month and 3 Months After a Suicide Attempt: A Comparison Between Two Samples. Arch. Suicide Res..

[48] Lizardi D., Stanley B. (2010). Treatment engagement: A neglected aspect in the psychiatric care of suicidal patients. Psychiatr. Serv..

[49] Michel K., Dey P., Stadler K., Valach L. (2004). Therapist Sensitivity towards Emotional Life-career Issues and the Working Alliance with Suicide Attempters. Arch. Suicide Res..

[50] Dunster-Page C., Haddock G., Wainwright L., Berry K. (2017). The relationship between therapeutic alliance and patient’s suicidal thoughts, self-harming behaviours and suicide attempts: A systematic review. J. Affect. Disord..

[51] Gysin-Maillart A., Schwab S., Soravia L., Megert M., Michel K. (2016). A Novel Brief Therapy for Patients Who Attempt Suicide: A 24-months Follow-Up Randomized Controlled Study of the Attempted Suicide Short Intervention Program (ASSIP). PLoS Med..

[52] Gysin-Maillart A.C., Soravia L.M., Gemperli A., Michel K. (2016). Suicide Ideation Is Related to Therapeutic Alliance in a Brief Therapy for Attempted Suicide. Arch. Suicide Res..

[53] Bryan C.J., Rozek D.C., Burch T.S., Leeson B., Clemans T.A. (2018). Therapeutic Alliance and Intervention Approach Among Acutely Suicidal Patients. Psychiatry.

[54] Alexander L.B., Luborsky L., Greenberg L.S., Pinsoff W.M. (1986). The Penn Helping Alliance Scales. The Psychotherapeutic Process: A Research Handbook.

[55] Hatcher R.L., Gillaspy J.A. (2006). Development and validation of arevised short version of the working alliance invento. Psychotherapy Res..

[56] Cooper J., Kapur N., Webb R., Lawlor M., Guthrie E., Mackway-Jones K., Appleby L. (2005). Suicide After Deliberate Self-Harm: A 4-Year Cohort Study. Am. J. Psychiatry.

[57] Inagaki M., Kawashima Y., Kawanishi C., Yonemoto N., Sugimoto T., Furuno T., Ikeshita K., Eto N., Tachikawa H., Shiraishi Y. (2015). Interventions to prevent repeat suicidal behavior in patients admitted to an emergency department for a suicide attempt: A meta-analysis. J. Affect. Disord..

[58] Morthorst B., Krogh J., Erlangsen A., Alberdi F., Nordentoft M. (2012). Effect of assertive outreach after suicide attempt in the AID (assertive intervention for deliberate self harm) trial: Randomised controlled trial. BMJ.

[59] Guthrie E., Kapur N., Mackway-Jones K., Chew-Graham C., Moorey J., Mendel E., Marino-Francis F., Sanderson S., Turpin C., Boddy G. (2001). Randomised controlled trial of brief psychological intervention after deliberate self poisoning Commentary: Another kind of talk that works?. BMJ.

[60] Brown G.K., Ten Have T., Henriques G.R., Xie S.X., Hollander J.E., Beck A.T. (2005). Cognitive therapy for the prevention of suicide attempts: A randomized controlled trial. JAMA.

[61] Linehan M.M., Comtois K.A., Murray A.M., Brown M.Z., Gallop R.J., Heard H.L., Korslund K.E., Tutek D.A., Reynolds S.K., Lindenboim N. (2006). Two-Year Randomized Controlled Trial and Follow-up of Dialectical Behavior Therapy vs Therapy by Experts for Suicidal Behaviors and Borderline Personality Disorder. Arch. Gen. Psychiatry.

[62] Rudd M.D., Bryan C.J., Wertenberger E.G., Peterson A.L., Young-McCaughan S., Mintz J., Williams S.R., Arne K.A., Breitbach J., Delano K. (2015). Brief Cognitive-Behavioral Therapy Effects on Post-Treatment Suicide Attempts in a Military Sample: Results of a Randomized Clinical Trial With 2-Year Follow-Up. Am. J. Psychiatry.

[63] Rudd M.D., Joiner T., Rajab M.H. (2001). Treating Suicidal Behavior: An Effective, Time-Limited Approach.

[64] Beck A.T., Salkovskis P. (1996). Beyond belief: A theory of modes, personality, and psychopathology. Frontiers of Cognitive Therapy.

[65] Rudd M.D. (2000). The suicidal mode: A cognitive-behavioral model of suicidality. Suicide Life-Threat. Behav..

[66] Linehan M.M., Bohart A.C., Greenberg L. (1997). Validation and psychotherapy. Empathy Reconsideration: New Directions in Psychotherapy.

[67] Rizvi S., Michel K., Jobes D.A. (2011). The Therapeutic Relationship in Dialectical Behaviour Therapy. Building a Therapeutic Alliance with the Suicidal Patient.

[68] Jobes D.A. (2006). Managing Suicidal Risk: A Collaborative Approach.

[69] Michel K., Gysin-Maillart A. (2015). ASSIP—Attempted Suicide Short Intervention Program: A Manual for Clinicians.

[70] Jobes D.A., Drozd J.F. (2004). The CAMS Approach to Working with Suicidal Patients. J. Contemp. Psychother..

[71] Rudd M.D., Joiner T., Brown G.K., Cukrowicz K., Jobes D.A., Silverman M., Cordero L. (2009). Informed consent with suicidal patients: Rethinking risks in (and out of) treatment. Psychotherapy.

[72] Weinberg I., Ronningstam E., Goldblatt M.J., Schechter M., Wheelis J., Maltsberger J.T. (2010). Strategies in treatment of suicidality: Identification of common and treatment-specific interventions in empirically supported treatment manuals. J. Clin. Psychiatry.

[73] Schiavone F.L., Links P.S. (2013). Common elements for the psychotherapeutic management of patients with Self Injurious Behavior. Child Abus. Negl..

[74] Ryan R.M., Deci E.L. (2000). Self-determination theory and the facilitation of intrinsic motivation, social development, and well-being. Am. Psychol..

[75] Michel K., Maltsberger J.T., Jobes D.A., Leenaars A.A., Orbach I., Stadler K., Dey P., Young R.A., Valach L. (2002). Discovering the Truth in Attempted Suicide. Am. J. Psychother..

[76] McCabe R., Garside R., Backhouse A., Xanthopoulou P. (2018). Effectiveness of brief psychological interventions for suicidal presentations: A systematic review. BMC Psychiatry.

[77] Meerwijk E.L., Parekh A., Oquendo M.A., Allen I.E., Franck L.S., Lee K.A. (2016). Direct versus indirect psychosocial and behavioural interventions to prevent suicide and suicide attempts: A systematic review and meta-analysis. Lancet Psychiatry.

[78] Michel K., Valach L. (1997). Suicide as goal-directed action. Arch. Suicide Res..

[79] Lippard E.T., Nemeroff C.B. (2020). The Devastating Clinical Consequences of Child Abuse and Neglect: Increased Disease Vulnerability and Poor Treatment Response in Mood Disorders. Am. J. Psychiatry.

[80] Lynch T.R., Chapman A.L., Rosenthal M.Z., Kuo J.R., Linehan M.M. (2006). Mechanisms of change in dialectical behavior therapy: Theoretical and empirical observations. J. Clin. Psychol..

[81] Wagner G., Li M., Sacchet M.D., Richard-Devantoy S., Turecki G., Bär K.-J., Gotlib I.H., Walter M., Jollant F. (2021). Functional network alterations differently associated with suicidal ideas and acts in depressed patients: An indirect support to the transition model. Transl. Psychiatry.

[82] Crowell S.E., Beauchaine T.P., Linehan M.M. (2009). A biosocial developmental model of borderline personality: Elaborating and extending linehan’s theory. Psychol. Bull..

[83] Reisch T., Seifritz E., Esposito F., Wiest R., Valach L., Michel K. (2010). An fMRI study on mental pain and suicidal behavior. J. Affect. Disord..

